# Supplementary material for: Impact of urbanisation on the gaps of hypertension prevalence, awareness and treatment among older age in China: a cross-sectional study
Source: BMJ Open. 2022 Jun 30;12(6):e057065. doi: 10.1136/bmjopen-2021-057065 (PMC9247652; doi:10.1136/bmjopen-2021-057065)
Supplement: Author's manuscript [file bmjopen-2021-057065.draft_revisions.pdf]

BMJ Open is committed to open peer review. As part of this commitment we make the peer review history of every article we publish publicly available.

When an article is published we post the peer reviewers' comments and the authors' responses online. We also post the versions of the paper that were used during peer review. These are the versions that the peer review comments apply to.

The versions of the paper that follow are the versions that were submitted during the peer review process. They are not the versions of record or the final published versions. They should not be cited or distributed as the published version of this manuscript.

BMJ Open is an open access journal and the full, final, typeset and author-corrected version of record of the manuscript is available on our site with no access controls, subscription charges or pay-per-view fees (http://bmjopen.bmj.com).

If you have any questions on BMJ Open's open peer review process please email info.bmjopen@bmj.com

BMJ Open

The Role of Urbanization in Impacting on the Urban-Rural Prevalence, Diagnosis and Medication of Hypertension Gaps in Older Age

Journal:	*BMJ Open*
Manuscript ID	bmjopen-2021-057065
Article Type:	Original research
Date Submitted by the Author:	03-Sep-2021

Complete List of Authors:	Yu, Qiutong; Shandong University,<br>Zuo, Genyong; Shandong University, Centre for Health Management and Policy Research, School of Public Health; Shandong University, NHC Key Laboratory of Health Economics and Policy Research

Keywords:	Hypertension < CARDIOLOGY, Health policy < HEALTH SERVICES ADMINISTRATION & MANAGEMENT, PUBLIC HEALTH

SCHOLARONE™ Manuscripts

I, the Submitting Author has the right to grant and does grant on behalf of all authors of the Work (as defined in the below author licence), an exclusive licence and/or a non-exclusive licence for contributions from authors who are: i) UK Crown employees; ii) where BMJ has agreed a CC-BY licence shall apply, and/or iii) in accordance with the terms applicable for US Federal Government officers or employees acting as part of their official duties; on a worldwide, perpetual, irrevocable, royalty-free basis to BMJ Publishing Group Ltd ("BMJ") its licensees and where the relevant Journal is co-owned by BMJ to the co-owners of the Journal, to publish the Work in this journal and any other BMJ products and to exploit all rights, as set out in our licence.

The Submitting Author accepts and understands that any supply made under these terms is made by BMJ to the Submitting Author unless you are acting as an employee on behalf of your employer or a postgraduate student of an affiliated institution which is paying any applicable article publishing charge ("APC") for Open Access articles. Where the Submitting Author wishes to make the Work available on an Open Access basis (and intends to pay the relevant APC), the terms of reuse of such Open Access shall be governed by a Creative Commons licence – details of these licences and which Creative Commons licence will apply to this Work are set out in our licence referred to above.

Other than as permitted in any relevant BMJ Author's Self Archiving Policies, I confirm this Work has not been accepted for publication elsewhere, is not being considered for publication elsewhere and does not duplicate material already published. I confirm all authors consent to publication of this Work and authorise the granting of this licence.

For peer review only

The Role of Urbanization in Impacting on the Urban-Rural Prevalence, Diagnosis and Medication of Hypertension Gaps in Older Age

Qiutong Yu[1,2], Genyong Zuo[1,2]

[1]Centre for Health Management and Policy Research, School of Public Health, Cheeloo College of Medicine Shandong University, 44 Wen-hua-xi Road, Jinan 250012, Shandong, China

[2]NHC Key Laboratory of Health Economics and Policy Research, Shandong University, 44 Wen-hua-xi Road, Jinan 250012, Shandong, China.

Correspondence to

Name Dr Genyong Zuo;

Postal address Shandong University, Jinan 250012, Shandong, China.

e-mail smartyong@sdu.edu.cn

telephone 13864066105

Keywords hypertension urbanization elderly

Word count 3532

Abstract

Objectives To examine the impact of urbanization on the prevalence, diagnosis, and medication of hypertension among elderly people in China.

Design This cross-sectional study uses data from the most recent nationally representative Chinese Longitudinal Healthy Longevity Survey, 2018.

Setting The study surveyed 22 provinces in China.

Participants This study surveyed 15874 respondents in China, after excluding 62 individuals under the age of 65, 41 individuals had technical problems, 4549 individuals were missing date, 870 individuals answered not occupied, and 493 individuals refused to answer, 9859 participants were included in the final analysis

Primary and secondary outcome measures Hypertension referred to SBP ≥ 140 mmHg or DBP ≥ 90 mmHg or self-reported history of hypertension, diagnosis referred to being diagnosed as hypertension by the hospital, medication referred to participants responding to taking antihypertensive medications. Multiple logistic regression and Blinder-Oaxaca

For peer review only

decomposition were employed to verify whether there were differences in the prevalence, diagnosis and medication of hypertension among the four types of urbanization adults.

Results Hypertension prevalence among semi-urbanized adults was lower than non-urbanized rural adults (OR=0.944; P<0.05) which may be caused by different demographic composition caused by urbanization population migration. The probability of diagnosis (OR=1.112; P<0.05) and medication (OR=1.128; P<0.001) of hypertension among non-urbanized rural adults were significantly lower than those of urban-born adults. Additionally, education level, family income, economic zone, health insurance benefits were significantly positively associated with the proportion of diagnosis and medication of hypertension.

Conclusions Our findings point to the role of urbanization that generate differences within demographic composition, education, regions and health insurance in shaping the distribution of prevalence, diagnosis and medication of hypertension. Our study suggests that public health programs and policies for chronic diseases should be adjusted reasonably along with the process of urbanization and should combine individual-centered strategies.

Strengths and limitations of this study

This study systematically explored the differences in the prevalence, diagnosis and medication of hypertension between urban and rural areas caused by urbanization. Our study can provide insights for countries which were undergoing rapid social transformation and urbanization to develop effective measures to reduce health disparity.

To our knowledge, this was the first cross-sectional study to explore the disparities in the prevalence of hypertension between urban and rural areas according to the difference in demographic composition caused by urbanization. And we used Blinder-Oaxaca decomposition to see to what extent a series of factors can explain the urban-rural differences in the prevalence, diagnosis, and medication of hypertension.

Limitation of this study includes the lack of inter-provincial urban-rural migration, the reasons for urbanization of residents and the impact of environmental pollution on hypertension, because the CLHLS data did not include the above data.

Introduction

Hypertension is a major risk factor for stroke, heart disease, and renal failure[1]. Approximately 7.3 million people died from hypertension every year in the world [2]. During

For peer review only

1975- 2015, the number of hypertensive patients increased from 594 million to more than 1.1 billion, mostly in low-and middle-income countries (LMICs) [3]. Hypertension is a silent killer which rarely develops symptoms at an early stage. However, it is dangerous to ignore hypertension, because elevated blood pressure increases the risk of chronic diseases, an early diagnosis and treatment is the key to reducing the risk [4]. In LMICs, the proportion of people who were diagnosed and treated was lower than in high income countries [5].

The Grand Challenges emphasized the need to study the impact of urbanization on hypertension [6]. One of the key drivers of urbanization in LMIC was rural-to-urban migration, which was always accompanied by a sharp increase in the prevalence of chronic diseases due to socio-economic development and changes in lifestyles, and inequalities in health care utilization between urban and rural areas [7]. Therefore, it was meaningful to assess the impact of rural-to-urban migration on hypertension owing to its huge public health consequences.

Particular in China. China was experiencing a period of rapid urbanization. The seventh national census report in 2020 showed that floating people was 375.18 million, an increase of 69.75% over 2010 [8]. Moreover, according to the results of a national cross-sectional survey during 2012-2015 showed that the prevalence of hypertension in the population over 60 years old was 53.2% [9]. Furthermore, due to the imbalance between urban and rural development, there were gaps in the diagnosis and medication of hypertension between rural and urban areas. Although, Chinese government had made great efforts to fill the gap of diagnosis and medication of hypertension by explicit of huge investment in human resources to improve the number of doctors in Township Health Center and many other public health programs [10]. However, the rural/urban disparities that concerning health services still existed [11].

The relationship between hypertension and urbanization has been extensively studied. Previous studies had assessed the relationship between urbanization and the prevalence, diagnosis, and treatment of hypertension, as measured by the socioeconomic status [7, 11, 12]. The results of those analyses highlighted education, income, and proportion of insured individuals of urban adults are significantly higher than those of rural adults, and these factors were significantly positively correlated in the diagnosis and medication of hypertension. Previous

For peer review only

analyses also estimated the time trend of the prevalence, diagnosis and medication of hypertension with the development of urbanization [13, 14]. Comparing the observed trends in the prevalence, diagnosis and medication of hypertension with urbanization can provide insights into the development of rural and urban areas and their respective impacts on hypertension.

In view of current researches, available reports and publications had largely examined and investigated hypertension and urbanization but the relationship between urbanization and prevalence, diagnosis, medication of hypertension in China has not been fully studied. First, most of the previous studies only based on hukou or current residence and did not show the characteristics of the floating population [15]. Second, although there was a study that used Blinder-Oaxaca decomposition to explain the urban-rural disparities in hypertension [13], most studies did not quantify accurately to what extent the gaps in prevalence, diagnosis, medication of hypertension can be explained by urban-rural differences. Therefore, in this study, the urbanization classification was used to show in detail the changes in socio-demographic characteristics caused by population mobility and Blinder-Oaxaca was used to evaluate the gaps in prevalence, diagnosis and medication of hypertension caused by urban and rural areas.

The main purpose of this study was to examine the following: (1) Whether there was a gap in prevalence, diagnosis, and medication of hypertension between urban-born and non-urbanized rural adults; (2) if so, what extent the gaps in prevalence, diagnosis, and medication of hypertension can be explained by urban-rural differences. As far as we know, this was the first study to comprehensively show the impact of rural-to-urban migration on the prevalence, diagnosis, and medication of hypertension based on Socio-demographic characteristics of the floating population in China. We hoped that the research results will provide insights for countries which were undergoing rapid social transformation and urbanization to develop effective measures to reduce health disparity and achieve healthy aging.

For peer review only

Methods

Sampling

For this study, we used secondary data which derived from the 2018 Chinese Longitudinal Healthy Longevity Survey (CLHLS), an ongoing cohort project from 1998. The CLHLS had conducted seven waves, covering individuals from 500 communities in 22 provinces in China. Multi-stage cluster sampling was used to sample individuals from each province. The 2018 survey wave included 15874 Chinese elderly individuals. Those who were younger than 65 years old (n=62), had technical problems (n=41), missed data (n=4549), not occupied (n=870) or refused (n=493) were excluded. In total 9859 individuals were eligible for this study, including 6201 hypertensive patients, 4239 hypertensive patients were diagnosed by the hospital, and 3605 took medication.

Variables

The main dependent variables of this study were prevalence, diagnosis and medication. we defined hypertension as having a mean SBP ≥ 140 mmHg or DBP ≥ 90 mmHg or self-reported history of hypertension [16]. Diagnosis was classified as yes if the respondent had been diagnosed by hospital with hypertension. Medication was classified as yes if the respondent took anti-hypertension drugs.

The key independent variables were urbanization types. Four types of urbanization are identified: (1) non-urbanized, meaning that individuals were born and lived in rural area, and hold the rural hukou all the time;（2）semi-urbanized, meaning that individuals were born in rural area but had moved into and lived in urban area while still holding the rural hukou; (3) fully-urbanized, meaning that individuals were born in rural area, had moved in and lived in urban area and obtained the urban hukou; (4) urban-born, meaning that individuals were born and lived in urban area, and hold the urban hukou all the time [17].

According to previous studies, we also included other risk factors including demographic characteristics (age, sex, marital status and geographical region), socioeconomic characteristics (education, income, BMI and medical insurance), lifestyle behavior (smoking, drinking and exercise). Age of participants was categorized into three groups which was the age standards revised by the WHO in 2015. elderly age is 60-75, senile age is 75-90 and long-livers are after 90 [18]. Details of each participant's marital status were including married and

For peer review only - http://bmjopen.bmj.com/site/about/guidelines.xhtml

living with spouse, never married/separated/widowed and divorced. We divided regions by economic belt: the eastern region (including Beijing, Tianjin, Hebei, Shanghai, Jiangsu, Zhejiang, Fujian, Shandong, Guangdong and Hainan provinces); the central region (including Shanxi, Anhui, Henan, Hubei and Hunan provinces); the western region (including Guangxi, Chongqing, Sichuan and Shaanxi provinces); the northeast region (including Liaoning, Jilin and Heilongjiang provinces). Education and income were continuous variables, representing years of schooling and total household income last year, respectively. BMI was calculated as weight in kilograms divided by height in meters squared and classified into overweight and obese (BMI≥24kg/m^2) and others (BMI≤24kg/m^2) [19]. Medical insurances were classified into urban resident basic medical insurance (URBMI)/urban employee basic medical insurance (UEBMI), new rural cooperative medical scheme insurance (NCMS) and others-including commercial medical insurance and government free medical insurance. Smoking and drinking were classified into current or not current, regular exercise was classified into yes or no.

Statistical analysis

Data were analyzed using STATA 15.0 for descriptive analysis, logistic regressions and Blinder-Oaxaca decomposition.

Descriptive statistical data of hypertension prevalence, diagnosis and medication among adults of four types of urbanization were reported proportionally, and the corresponding chi-square and p-values were used to check whether there were statistically significant differences among adults of four urbanization levels. Continuous variables in demography, socioeconomic, and lifestyle behavior were estimated by means of averages, and categorical variables by proportions. The chi-square test was used for dichotomous variable, the t-test was used for continuous variables.

We used logistic regression to examine differences among adults of four types of urbanization in a multivariate analysis that adjusted for confounding variables. Adjusted odds ratios and their p values were reported. The model was specified as follows:

$$logit(P_i) = \ln(\frac{P_i}{1-P_i})$$
$$= \beta_0 + \beta_1 urbanization_i + \sum_{1}^{n} a_n X_{ni} \quad (1)$$

For peer review only - http://bmjopen.bmj.com/site/about/guidelines.xhtml

Where P_i represented the probability of prevalence, diagnosis, and medication of hypertension. $ubanization_i$ represented the four types of urbanization: non-urbanized, semi-urbanized, fully-urbanized and urban-born. β_0 was a constant term, coefficients β_1 represented disparities among four types of urbanization, X_{ni} were control variables.

Blinder-Oaxaca decomposition is a commonly used disparities decomposition method. Based on the above-mentioned Logit regression, this study will further use Blinder-Oaxaca decomposition to decompose the disparities in the prevalence, diagnosis, and medication of hypertension between urban and rural areas into three parts: endowment effect, coefficients effect, and interaction effect (The effect caused by the endowment effect and the coefficient effect at the same time) [20,21].

The factors that affect the prevalence, diagnosis, and treatment functions of hypertension were estimated for the two sets of samples, and the regression coefficients of the estimated equation are obtained. Oaxaca-Blinder decompose the differences between the two as follows:

$$\bar{y}_a - \bar{y}_b = (\bar{x}_a - \bar{x}_b)\beta_b + \bar{x}_b(\beta_a - \beta_b) + (\bar{x}_a - \bar{x}_b)(\beta_a - \beta_b) \quad (2)$$

Where α were the adults living in the urban areas, b were the adults living in the rural areas. $(\bar{x}_a - \bar{x}_b)\beta_b$ was called the "endowments effect" (or "explained component"); $\bar{x}_b(\beta_a - \beta_b)$ was called "coefficients effect" (or "the unexplained component"). $(\bar{x}_a - \bar{x}_b)(\beta_a - \beta_b)$ was the part that can be explained by the interaction of endowment effect and coefficient effect.

Patient and public involvement

No patient involved.

Results

Descriptive results

The background characteristics of the study participants were presented in Table 1. The prevalence, diagnosis and medication of hypertension in rural-born adults were significantly lower than those in urban-born adults, and the significance level at P <0.001. Descriptive results showed that non-urbanized, semi-urbanized, fully-urbanized and urban-born adults also differed significantly in all control variables (p<0.001), The years of education increased with the level of urbanization. The income of people born in cities was more than twice that

For peer review only

of those born in rural areas. The BMI of people born in cities was the highest, and the BMI increased with the level of urbanization. The proportion of rural-born adults participating in the New Cooperative Medical System accounted for approximately 70.2% of the sample.

Table 1 Background characteristics of study participants aged 65 and over from Chinese Longitudinal Healthy Longevity Survey 2018 survey data (n=9859)

Variables	Rural-born			Urban-born	Total	P
	Non-urbanized	semi-urbanized	Fully-urbanized			
Prevalence, n (%)						<0.001
No	1546 (37.37)	1149 (40.29)	494 (35.11)	469 (32.06)	3658(37.10)	
Yes	2591 (62.63)	1703 (59.71)	913 (64.89)	994 (67.94)	6201(62.90)	
Diagnosis, n (%)						<0.001
No	436 (21.21)	265 (19.46)	106 (13.15)	72(8.05)	879 (17.17)	
Yes	1620 (78.79)	1097 (80.54)	700 (86.85)	822 (91.95)	4239 (82.83)	
Medication, n (%)						<0.001
No	717 (35.13)	462 (34.20)	178 (22.17)	128 (14.30)	1485 (29.17)	
Yes	1324 (64.87)	889 (65.80)	625 (77.83)	767 (85.70)	3605 (70.83)	
Age, n (%)						<0.001
Elderly age	1045 (25.26)	689 (24.16)	257 (18.27)	467 (31.92)	2458 (24.93)	
Senile age	1491 (36.04)	1008 (35.34)	546 (38.81)	550 (37.59)	3595 (36.46)	
Long-livers	1601 (38.70)	1155 (40.50)	604 (42.93)	446 (30.49)	3806(38.60)	
Sex, n (%)						<0.001
Female	2403 (58.09)	1611 (56.49)	708 (50.32)	761 (52.02)	5483 (55.61)	
Male	1734 (41.91)	1241 (43.51)	699 (49.68)	702 (47.98)	4376 (44.39)	
Marital status[a], n (%)						<0.001
Not married	2401 (58.04)	1694 (59.40)	793 (56.36)	713 (48.74)	5601 (56.81)	
Married	1736 (41.96)	1158 (40.60)	614 (43.64)	750 (51.26)	4258 (43.19)	
Area, n (%)						<0.001
Eastern	1867 (45.13)	1198 (42.01)	762 (54.16)	1009(68.97)	4836 (49.05)	
Northeastern	118(2.85)	56(1.96)	143 (10.16)	121 (8.27)	438 (4.44)	
Central	1170 (28.28)	715 (25.07)	202 (14.36)	136 (9.30)	2223 (22.55)	
Western	982 (23.74)	883 (30.96)	300 (21.32)	197 (13.47)	2362 (23.96)	
Education, mean (SD)[b]	2.23(3.10)	2.17(3.12)	5.09(5.00)	7.72(5.21)	3.44(4.32)	<0.001
Income, mean (SD)	31000.21 (32620.93)	32741.37 (32764.62)	65050.23 (33761.20)	72712.83 (31715.16)	42553.08 (36874.99)	<0.001
BMI						<0.001
<24	2969 (71.77)	2057 (72.12)	862 (61.27)	845 (57.76)	6733 (68.29)	
≥24	1168 (28.23)	795 (27.88)	545 (38.73)	618 (42.24)	3126 (31.71)	
Somking, n (%)						<0.001
Not current	3468 (83.83)	2355 (82.57)	1257 (89.34)	1291(88.24)	8371(84.91)	

For peer review only

Current	669 (16.17)	497 (17.43)	150 (10.66)	172 (11.76)	1488 (15.09)	
Drinking, n (%)						0.030
Not current	3508 (84.80)	2420 (84.85)	1224 (86.99)	1277(87.29)	8429 (85.50)	
Current	629 (15.20)	432 (15.15)	183 (13.01)	186 (12.71)	1430 (14.50)	
Regular exercise, n (%)						<0.001
No	2911 (70.36)	1896 (66.48)	545 (38.73)	551 (37.66)	5903 (59.87)	
Yes	1226 (29.64)	956 (33.52)	862 (61.27)	912 (62.34)	3956 (40.13)	
medical insurance[c], n (%)						
None	342(8.27)	251(8.80)	260 (18.48)	254 (17.36)	1107 (11.23)	
UEBMI/URBMI	249(6.02)	298 (10.45)	829 (58.92)	989 (67.60)	2365 (23.99)	<0.001
NCMS	3472 (83.93)	2255 (79.07)	167 (11.87)	87(5.95)	5981 (60.67)	
Others	74(1.79)	48(8.80)	151 (10.73)	133(9.09)	406(4.12)	

[a] Not married: never married, separated, widowed and divorced; Married: married and living with spouse/cohabiting.

[b] SD: Standard deviation

[c] UEBMI/URBMI: urban resident basic medical insurance /urban employee basic medical insurance; NCMS: new rural cooperative medical scheme insurance; Others: commercial medical insurance and government free medical insurance.

Logistic regression analysis

We regressed the three binary variables (prevalence, diagnosis and medication) on demographic characteristics, socioeconomic characteristic, lifestyle behavior in Table 2. After controlling for confounding variables, semi-urbanized adults had lower odds of hypertension than non-urbanized adults (OR=0.094; P<0.05). Urban-born adults were 11.2% (OR=1.112; P<0.05) times more likely to be diagnosed than the reference group (rural-born). There was similar difference in medication, urban-born adults were 12.8% (OR=1.128; P<0.001) times more likely to be medicated than non-urbanized adults.

The risk of hypertension was 11.7% (OR=1.117; P<0.001) times more likely among senile age than reference group (elderly age). Long-livers were less likely to be diagnosed (0R=0.85; P<0.001) and be medicated (OR=0.859; P<0.001) than elderly age. The association between geographical region and hypertension was significant difference. Specifically, participants living in Central and Western China had 88.9% (OR=0.889; P<0.001) ,93.1% (OR=0.931; P<0.001) lower odds of hypertension in the Eastern China, respectively.

For peer review only

Especially for adults with UEBMI /URBMI had a higher probability of diagnosis (OR=1.667; P<0.001) and medication (OR=1.327; P<0.05).

Table 2 Multivariate logistic regression model to determine factors associated with prevalence, diagnosis, medication among participants aged 65 years and above

Variables	Prevalence AOR	Diagnosis AOR	Medication Use AOR
semi-urbanized	0.944*	1.045	1.011
	(0.024)	(0.047)	(0.039)
Fully-urbanized	0.980	1.048	1.071
	(0.027)	(0.053)	(0.045)
Urban-born	0.995	1.112*	1.128***
	(0.022)	(0.048)	(0.040)
Age: Senile age	1.117***	1.019	1.085
	(0.033)	(0.056)	(0.049)
Age: Long-livers	0.976	0.850***	0.859***
	(0.022)	(0.035)	(0.029)
Sex: Male	0.843***	0.837	0.731***
	(0.043)	(0.077)	(0.057)
Married a	0.909	1.066	1.097
	(0.048)	(0.100)	(0.087)
Region: Northeastern	0.958	0.813*	0.873
	(0.052)	(0.078)	(0.072)
Region: Central	0.889***	0.976	0.954
	(0.016)	(0.033)	(0.027)
Region: Western	0.931***	0.903***	0.889***
	(0.013)	(0.021)	(0.018)
Education	1.007	1.030*	1.034**
	(0.007)	(0.013)	(0.011)
Income	1.000	1.000**	1.000***
	(0.000)	(0.000)	(0.000)
BMI≥24	2.087***	1.438***	1.464***
	(0.106)	(0.125)	(0.104)
Somking: Current	0.937	1.073	1.041
	(0.061)	(0.129)	(0.105)
Drinking: Current	0.990	0.676***	0.708***
	(0.064)	(0.077)	(0.070)
Regular exercise: Yes	1.079	1.004	1.085
	(0.051)	(0.084)	(0.077)
UEBMI /URBMI [b]	1.135	1.667***	1.327*
	(0.092)	(0.250)	(0.167)
NCMS [c]	1.067	1.035	0.976
	(0.085)	(0.141)	(0.110)
Others [d]	0.975	1.100	0.957

For peer review only

	(0.115)	(0.322)	(0.170)
Constants	0.000***	0.000***	0.000***
	(0.155)	(0.716)	(0.317)
Conservation	9859	5118	5090

Robust standard errors are reported in parenthesis. *** $p < 0.001$, ** $p < 0.01$, * $p < 0.05$

[a] Married: married and living with spouse/cohabiting.

[b] UEBMI/URBMI: urban resident basic medical insurance /urban employee basic medical insurance;

[c] NCMS: new rural cooperative medical scheme insurance;

[d] Others: commercial medical insurance and government free medical insurance.

Decomposition analysis

The logistic regression analysis in Table 2 had confirmed the urban-rural disparities in the prevalence, diagnosis and medication of hypertension. For further quantified the observed factor contribution behind the disparities, we used the Blinder-Oaxaca decomposition. In Table 3, the decomposition results showed that the probabilities of being hypertensive were 63.09% for urban adults and 62.63% for rural adults. Both the coefficients effect and interaction effect were significant. Results for diagnosis and medication were somewhat different. The total difference between urban and rural adults was 6.74% and 9.94%, respectively. Only the endowments effect was significant, accounted for 5.88% and 10.12% of the total difference, respectively.

Table 3 Blinder-Oaxaca decomposition results between urban and rural adults

	Prevalence	Diagnosis	Medication
Predicted probability			
urban	63.09%***	85.53%***	74.81%***
	(0.0064)	(0.0064)	(0.0079)
rural	62.63%***	78.79%***	64.87%***
	(0.0075)	(0.0091)	(0.0106)
Difference in predicted probability			
Total difference	0.50%	6.74%***	9.94%***
	(0.0098)	(0.0111)	(0.0132)
Due to endowments (explained)	0.74%	5.88%***	10.12%***
	(0.0121)	(0.0165)	(0.0193)
Due to coefficients (unexplained)	-2.88%*	1.98%	2.16%
	(0.0111)	(0.0125)	(0.0148)
Due to interaction	2.60%*	-1.12%	-2.34%
	(0.0131)	(0.0175)	(0.0204)

For peer review only

Robust standard errors are reported in parenthesis. *** $p < 0.001$, ** $p < 0.01$, * $p < 0.05$

Discussion

Finding from our study of 9859 adults in 22 provinces suggested that urbanization was associated with prevalence, diagnosis and medication of hypertension. The prevalence of hypertension in semi-urbanized adults was lower than that in rural adults. The proportion of diagnosis and medication of hypertension in non-urbanized rural adults were lower than those in urban-born adults. Our study included 22 diverse provinces in China, this sample was representative of the entire Chinese population.

To our knowledge, we were the first cross-sectional study to explore the disparities in the prevalence of hypertension between urban and rural areas according to the difference in demographic composition caused by urbanization. The prevalence of hypertension in semi-urbanized adults was lower than that in rural adults. This was highly relevant given that a large portion of floating people that migrating from rural to urban were young laborers[22], the elderly is no exception. Most of the migrants are young and healthy elderly, in order to provide childcare [23]. Blinder-Oaxaca decomposition result was slightly different from the Logistic regression results because that was based on the location of adults living in urban and rural areas, which showed the need for research on the floating population. Although some studies have examined hypertension prevalence that correlated with urbanization [12, 24], these researches did not show in detail the differences in demographic composition caused by urbanization. By contrast, our study was based on socio-demographic characteristics of the floating population suggesting that the proportion of young elderly among semi-urbanized adults was large, so the prevalence of hypertension was low.

We also observed a significant association between diagnosis, medication of hypertension and the segment of education in urbanization. Although some studies had documented a positive impact of education on diagnosis and medication of hypertension [25, 26], few study considered the impact of education in the context of urbanization. We had concluded that the higher the level of urbanization, the higher the level of education. As better education levels often correlated with better family income. People with better education level may pay more attention to their health [27]. Thus, they may have higher health care expenditure budget to seek health care services of high quality. Therefore, improving

population education level, especially rural adults, may have important practical and theoretical significance.

The influence of urbanization on diagnosis and medication of hypertension seemed different in regions in China. According to the results, adults living in eastern China had a higher proportion of diagnosis and medication of hypertension than that of central and western China. The positive association with diagnosis and medication in China seems to be in accordance with findings of other studies conducted [13, 28, 29]. The economy of the eastern China was more developed and the level of urbanization was higher than that of the central and western regions [30,31]. Thus, adults living in eastern China seem to have acquired more community health facilities, social and economic resources brought by urbanization that had effect on health [32]. Although there were strong evidences that the proportion of diagnosis and medication of hypertension in rural areas in the Northeast and other regions were lower than in urban areas [33, 34], these had not compared the disparities between regions of China. All in all, higher urbanization level may help people living in eastern China experienced better social environment and health services, thereby achieving heathy aging.

Distinct insurance difference could observe in our research. Urban insurance enrollees had higher diagnosis and medication than rural insurance enrollees and uninsured adults, which was consistent with earlier findings [35]. However, what kind of medical insurance the adults participate in depends on China's unique Household Registration System (HRS, Hukou System), in which most rural-to-urban migrants can only participate in the new rural cooperative medical scheme insurance. In other words, these rural-to-urban migrants enrolled in NCMS of hometown always had to return for medical care to receive full reimbursement [36]. In addition, previous studies had shown that participating in local medical insurance for migrants was more conducive to the management and control of hypertension [37, 38]. Therefore, further efforts were needed to integrate the health insurance system into one uniform system in a propriate way which was beneficial the management of hypertension and reduced obstacles in the process of urbanization. We did not observe any association with commercial medical insurance and government free medical insurance; because, there were only 406 other medical insurance cases documented in our study.

For peer review only

Limitations

There were several limitations. First, this study did not include inter-provincial urban-rural migration, such as migration from rural areas in western China to rural areas in eastern China. Due to regional differences, the diagnosis and medication of hypertension may be different. Second, our research did not include the reasons for urbanization migration, people who migrated for different reasons had different socioeconomic status, psychological and social pressures, and different health condition. Third, environmental pollution, such as the impact of air and noise pollution on the prevalence of hypertension was not included in this study. The above factors cannot be studied in the research because they were not included in the CLHLS questionnaire. Therefore, researchers can carry out relevant research in the future.

Conclusion

Urbanization was significantly associated with prevalence, diagnosis and medication of hypertension, especially between non-urbanized rural adults and urban-born adults, in a representative national population in China. Our findings point to the role of urbanization that generate differences within demographic composition, education, regions and health insurance in shaping the distribution of prevalence, diagnosis and medication of hypertension. Our study suggests that public health programs and policies for chronic diseases should be adjusted reasonably along with the process of urbanization and combine individual-centered strategies.

Abbreviations CLHLS: Chinese Longitudinal Healthy Longevity Survey; LMIC: low- and middle-income countries; BMI: body mass index; URBMI: urban resident basic medical insurance; UEBMI: urban employee basic medical insurance; NCMS: new rural cooperative medical scheme insurance； SD: Standard deviation

Acknowledgements We thank the Chinese Longitudinal Healthy Longevity Survey, which provided the data in this research.

For peer review only

Contributions QY: conceptualization, methodology, data analysis, and writing. GZ: methodology, conceptualization, writing with review, funding. All authors approved the final version of the paper.

Funding This study was supported by National Natural Science Foundation of China (71774102).

Patient consent Not required.

Provenance and peer review Not commissioned.

Data availability statement Data are available in open research data service platform of Peking University. Data for this study were sourced from Chinese Longitudinal Healthy Longevity Survey (CLHLS) and available here: https://opendata.pku.edu.cn

Ethics approval and consent to participate The CLHLS was approved by the Ethical Review Committee of Peking University (IRB00001052–13074). All participants signed the informed consent at the time of participation. The research has been performed in accordance with the Declaration of Helsinki.

Competing interests

The authors declare that they have no competing interests.

ORCID IDs

Qiutong Yu http://orcid.org/0000-0002-1758-0692

Genyong Zuo http://orcid.org/0000-0002-9305-9671

PREFERRENCES

1 Stanaway JD, Afshin A, Gakidou E, *et al*. Global, regional, and national comparative risk assessment of 84 behavioural, environmental and occupational, and metabolic risks or clusters of risks for 195 countries and territories, 1990 - 2017: a systematic analysis for the Global Burden of Disease Study 2017. *The Lancet* 2018;392(10159):1923-94.

For peer review only

2 World Health Organization. *Hypertension and older people*. Geneva: World Health Organization, 2017.
3 Zhou B, Bentham J, Di Cesare M, *et al*. Worldwide trends in blood pressure from 1975 to 2015: a pooled analysis of 1479 population-based measurement studies with 19 • 1 million participants. *The Lancet* 2017;389(10064):37-55.
4 Zhou B, Danaei G, Stevens GA, *et al*. Long-term and recent trends in hypertension awareness, treatment, and control in 12 high-income countries: an analysis of 123 nationally representative surveys. *The Lancet* 2019;394(10199):639-51.
5 Ikeda N, Sapienza D, Guerrero R, *et al*. Control of hypertension with medication: a comparative analysis of national surveys in 20 countries. Bull World Health Organ 2014;92(1):10-19C.
6 Daar AS, Singer PA, Persad DL, *et al*. Grand challenges in chronic non-communicable diseases. *Nature* 2007;450(7169):494-6.
7 Toshiaki A MH. Urbanization and Inequality in Hypertension Diagnosis and Medication in Indonesia. Japan： Asian Development Bank Institute, 2016.
8 National Bureau of Statistics of the People's Republic of China. Interpretation of the Seventh National Census Communiqué, 2021. Available http://www.stats.gov.cn/tjsj/sjjd/202105/t20210512_1817336.html.
9 Hua Q, Fan L, Li J, *et al*. 2019 Chinese guideline for the management of hypertension in the elderly. *J Geriatr Cardiol* 2019;16(2):67-99.
10 Hesketh T, Zhou X. Hypertension in China: the gap between policy and practice. *The Lancet* 2017;390(10112):2529-30.
11 Aizawa T. Inequality in the treatment of diabetes and hypertension across residency status in China. *Ethn Health* 2021;26(4):512-29.
12 Xiaohui H. Urban－Rural Disparity of Overweight, Hypertension, Undiagnosed Hypertension, and Untreated Hypertension in China. *Asia-Pacific Journal Of Public Health* 2008;20:159-69.
13 Li J, Shi L, Li S, *et al*. Urban-rural disparities in hypertension prevalence, detection, and medication use among Chinese Adults from 1993 to 2011. *Int J Equity Health* 2017;16(1):50.
14 Xing L, Jing L, Tian Y, *et al*. Urban-Rural disparities in status of hypertension in northeast China: a population-based study, 2017-2019. *Clin Epidemiol* 2019;11:801-20.
15 Bernabe-Ortiz A, Sanchez JF, Carrillo-Larco RM, *et al*. Rural-to-urban migration and risk of hypertension: longitudinal results of the PERU MIGRANT study. *J Hum Hypertens* 2017;31(1):22-28.
16 World Health Organization. A global brief on hypertension: silent killer, global public health crisis: World Health Day 2013, 2013. Available: https://apps.who.int/iris/handle/10665/79059
17 Fan Y, VW, QL. Childhood adversities, urbanization and depressive symptoms among middle-aged and older adults: evidence from a national survey in China. *Ageing & Society* 2015;36:1031-51.
18 Dyussenbayev A. Age Periods Of Human Life. *Advances in Social Sciences Research Journal* 2017;4:258-63.
19 Chittaranjan S, Yajnik JSY. Appropriate body-mass index for Asian populations and its implications for policy and intervention strategies. *The Lancet* 2004;363(9403):157-63.
20 Blinder AS. Wage Discrimination: Reduced Form and Structural Estimates. *The Journal of Human Resources* 1973;8:436-55.

21 Oaxaca R. Male-Female Wage Differentials in Urban Labor Markets. *International Economic Review* 1973;14:693-709.
22 Zhang W. A forecast analysis on world population and urbanization process. *Environment, Development and Sustainability* 2007;10(6):717-30.
23 Cheng Y, Rosenberg M, Winterton R, *et al*. Mobilities of Older Chinese Rural-Urban Migrants: A Case Study in Beijing. *Int J Environ Res Public Health* 2019;16(3)
24 Ahmed S, Tariqujjaman M, Rahman MA, *et al*. Inequalities in the prevalence of undiagnosed hypertension among Bangladeshi adults: evidence from a nationwide survey. *Int J Equity Health* 2019;18(1):33.
25 Daskalopoulou SS, Rabi DM, Zarnke KB, *et al*. The 2015 Canadian Hypertension Education Program recommendations for blood pressure measurement, diagnosis, assessment of risk, prevention, and treatment of hypertension. *Can J Cardiol* 2015;31(5):549-68.
26 Kaberi D RP, Luc P. BP, Robert R. Q. Managing hypertension: evidence supporting the 2013/2014 recommendations of the Canadian Hypertension Education Program. *CMAJ* 2015:116-19.
27 Leng B, Jin Y, Li G, *et al*. Socioeconomic status and hypertension: a meta-analysis. *J Hypertens* 2015;33(2):221-9.
28 Lu J, Lu Y, Wang X, *et al*. Prevalence, awareness, treatment, and control of hypertension in China: data from 1・7 million adults in a population-based screening study (China PEACE Million Persons Project). *The Lancet* 2017;390(10112):2549-58.
29 Kristi R DG, Paul M, Xigui W, *et al*. Geographic variations in the prevalence, awareness, treatment and control of hypertension in China. *Journal of Hypertension* 2003;21:1273-80.
30 Yu X, Zhang W. All-cause mortality rate in China: do residents in economically developed regions have better health? *Int J Equity Health* 2020;19(1):12.
31 Maimaitiming A, Xiaolei Z, Huhua C. Urbanization in Western China. *Chinese Journal of Population Resources and Environment* 2013;11(1):79-86.
32 Liu W, Liu Y, Twum P, *et al*. National equity of health resource allocation in China: data from 2009 to 2013. *Int J Equity Health* 2016;15:68.
33 Ma WJ, Tang JL, Zhang YH, *et al*. Hypertension prevalence, awareness, treatment, control, and associated factors in adults in southern China. *Am J Hypertens* 2012;25(5):590-6.
34 Zhao L, Sun W, Wang J, *et al*. Differences in the treatment and control of hypertension in urban and rural residents of the northeastern region of the People's Republic of China: a cross-sectional study. *Clin Exp Hypertens* 2019;41(4):366-72.
35 Feng L. Undiagnosed and Uncontrolled Chronic Conditions in China: Could Social Health Insurance Consolidation Make a Change? *Medical Care Research and Review* 2017;75:479-515.
36 Peiyuan Q YY, Juying Z and Xiao M. Rural-to-urban migration and its implication fornew cooperative medical scheme coverage and utilization in China. *BMC Public Health* 2011;11
37 Fang H, Jin Y, Zhao M, et al. Does Migration Limit the Effect of Health Insurance on Hypertension Management in China? *Int J Environ Res Public Health* 2017;14(10)
38 Li H, Zhu W, Xia H, et al. Cross-Sectional Study on the Management and Control of Hypertension Among Migrants in Primary Care: What Is the Impact of Segmented Health Insurance Schemes? *J Am Heart Assoc* 2019;8(16):e012674.

For peer review only

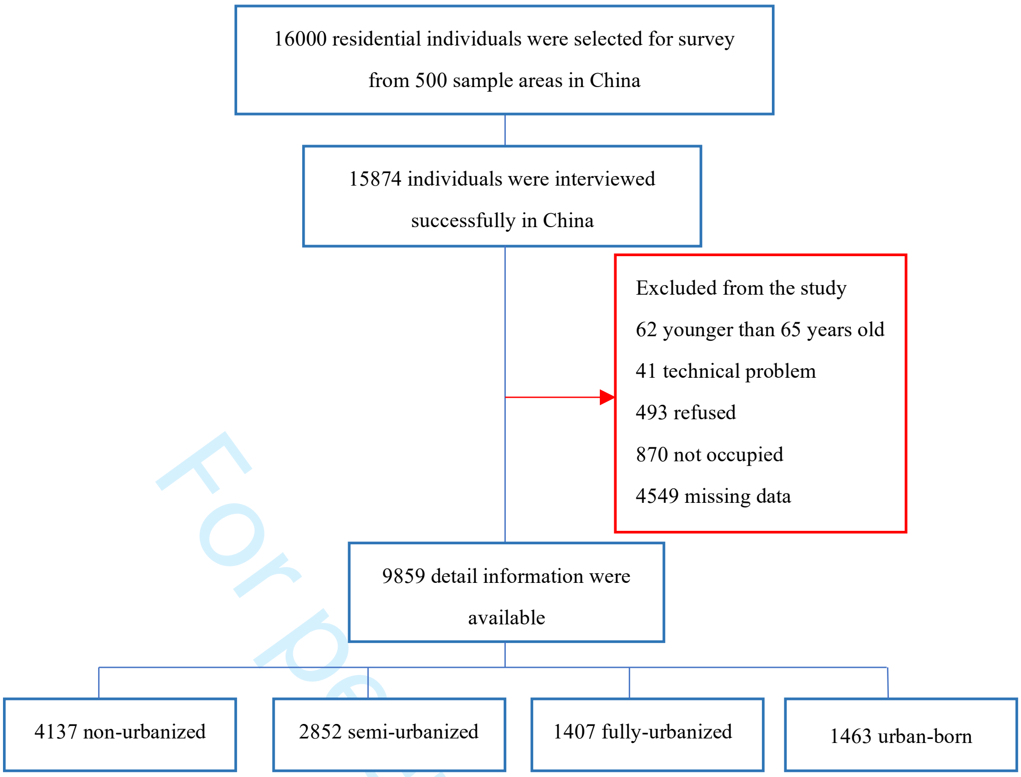

Figure1 A flowchart for sample selection

For peer review only

STROBE Statement—Checklist of items that should be included in reports of ***cross-sectional studies***

	Item No	Recommendation	Page No
**Title and abstract**	1	(*a*) Indicate the study's design with a commonly used term in the title or the abstract	1
		(*b*) Provide in the abstract an informative and balanced summary of what was done and what was found	1
**Introduction**			
Background/rationale	2	Explain the scientific background and rationale for the investigation being reported	2-4
Objectives	3	State specific objectives, including any prespecified hypotheses	4
**Methods**			
Study design	4	Present key elements of study design early in the paper	5
Setting	5	Describe the setting, locations, and relevant dates, including periods of recruitment, exposure, follow-up, and data collection	5
Participants	6	(*a*) Give the eligibility criteria, and the sources and methods of selection of participants	5
Variables	7	Clearly define all outcomes, exposures, predictors, potential confounders, and effect modifiers. Give diagnostic criteria, if applicable	5-6
Data sources/ measurement	8*	For each variable of interest, give sources of data and details of methods of assessment (measurement). Describe comparability of assessment methods if there is more than one group	*6*
Bias	9	Describe any efforts to address potential sources of bias	5
Study size	10	Explain how the study size was arrived at	5
Quantitative variables	11	Explain how quantitative variables were handled in the analyses. If applicable, describe which groupings were chosen and why	6
Statistical methods	12	(*a*) Describe all statistical methods, including those used to control for confounding	6
		(*b*) Describe any methods used to examine subgroups and interactions	7
		(*c*) Explain how missing data were addressed	5
		(*d*) If applicable, describe analytical methods taking account of sampling strategy	
		(*e*) Describe any sensitivity analyses	8

For peer review only - http://bmjopen.bmj.com/site/about/guidelines.xhtml

**Results**			
Participants	13*	(a) Report numbers of individuals at each stage of study—eg numbers potentially eligible, examined for eligibility, confirmed eligible, included in the study, completing follow-up, and analysed	5
		(b) Give reasons for non-participation at each stage	
		(c) Consider use of a flow diagram	Image
Descriptive data	14*	(a) Give characteristics of study participants (eg demographic, clinical, social) and information on exposures and potential confounders	7
		(b) Indicate number of participants with missing data for each variable of interest	5
Outcome data	15*	Report numbers of outcome events or summary measures	5
Main results	16	(*a*) Give unadjusted estimates and, if applicable, confounder-adjusted estimates and their precision (eg, 95% confidence interval). Make clear which confounders were adjusted for and why they were included	6
		(*b*) Report category boundaries when continuous variables were categorized	9
		(*c*) If relevant, consider translating estimates of relative risk into absolute risk for a meaningful time period	
Other analyses	17	Report other analyses done—eg analyses of subgroups and interactions, and sensitivity analyses	11
**Discussion**			
Key results	18	Summarise key results with reference to study objectives	
Limitations	19	Discuss limitations of the study, taking into account sources of potential bias or imprecision. Discuss both direction and magnitude of any potential bias	12-13
Interpretation	20	Give a cautious overall interpretation of results considering objectives, limitations, multiplicity of analyses, results from similar studies, and other relevant evidence	14
Generalisability	21	Discuss the generalisability (external validity) of the study results	14
**Other information**			
Funding	22	Give the source of funding and the role of the funders for the present study and, if applicable, for the original study on which the present article is based	15

For peer review only - http://bmjopen.bmj.com/site/about/guidelines.xhtml

*Give information separately for exposed and unexposed groups.

Note: An Explanation and Elaboration article discusses each checklist item and gives methodological background and published examples of transparent reporting. The STROBE checklist is best used in conjunction with this article (freely available on the Web sites of PLoS Medicine at http://www.plosmedicine.org/, Annals of Internal Medicine at http://www.annals.org/, and Epidemiology at http://www.epidem.com/). Information on the STROBE Initiative is available at www.strobe-statement.org.

For peer review only

For peer review only - http://bmjopen.bmj.com/site/about/guidelines.xhtml

BMJ Open

Impact of Urbanization on the Gaps of Hypertension Prevalence, Awareness, and Treatment among Older Age in China: A Cross-Sectional Study

Journal:	*BMJ Open*
Manuscript ID	bmjopen-2021-057065.R1
Article Type:	Original research
Date Submitted by the Author:	07-Mar-2022

Complete List of Authors:	Yu, Qiutong; Shandong University, Centre for Health Management and Policy Research, School of Public Health; Shandong University, NHC Key Laboratory of Health Economics and Policy Research<br>Zuo, Genyong; Shandong University, Centre for Health Management and Policy Research, School of Public Health; Shandong University, NHC Key Laboratory of Health Economics and Policy Research

**Primary Subject Heading**:	Public health
Secondary Subject Heading:	Global health, Cardiovascular medicine
Keywords:	Hypertension < CARDIOLOGY, Health policy < HEALTH SERVICES ADMINISTRATION & MANAGEMENT, PUBLIC HEALTH

SCHOLARONE™
Manuscripts

I, the Submitting Author has the right to grant and does grant on behalf of all authors of the Work (as defined in the below author licence), an exclusive licence and/or a non-exclusive licence for contributions from authors who are: i) UK Crown employees; ii) where BMJ has agreed a CC-BY licence shall apply, and/or iii) in accordance with the terms applicable for US Federal Government officers or employees acting as part of their official duties; on a worldwide, perpetual, irrevocable, royalty-free basis to BMJ Publishing Group Ltd ("BMJ") its licensees and where the relevant Journal is co-owned by BMJ to the co-owners of the Journal, to publish the Work in this journal and any other BMJ products and to exploit all rights, as set out in our licence.

The Submitting Author accepts and understands that any supply made under these terms is made by BMJ to the Submitting Author unless you are acting as an employee on behalf of your employer or a postgraduate student of an affiliated institution which is paying any applicable article publishing charge ("APC") for Open Access articles. Where the Submitting Author wishes to make the Work available on an Open Access basis (and intends to pay the relevant APC), the terms of reuse of such Open Access shall be governed by a Creative Commons licence – details of these licences and which Creative Commons licence will apply to this Work are set out in our licence referred to above.

Other than as permitted in any relevant BMJ Author's Self Archiving Policies, I confirm this Work has not been accepted for publication elsewhere, is not being considered for publication elsewhere and does not duplicate material already published. I confirm all authors consent to publication of this Work and authorise the granting of this licence.

For peer review only

Impact of Urbanization on the Gaps of Hypertension Prevalence, Awareness, and Treatment among Older Age in China: A Cross-Sectional Study

Qiutong Yu[1,2], Genyong Zuo*[1,2]

[1]Centre for Health Management and Policy Research, School of Public Health, Cheeloo College of Medicine Shandong University, 44 Wen-hua-xi Road, Jinan 250012, Shandong, China

[2]NHC Key Laboratory of Health Economics and Policy Research, Shandong University, 44 Wen-hua-xi Road, Jinan 250012, Shandong, China.

Correspondence to Professor Genyong Zuo

Postal address Shandong University, Jinan 250012, Shandong, China.

E-mail smartyong@sdu.edu.cn;

Keywords hypertension urbanization elderly

Word count 3967

Abstract

Objectives To examine the impact of urbanization on the prevalence, awareness, and treatment of hypertension among elderly in China.

Design This cross-sectional study used data from the most recent nationally representative Chinese Longitudinal Healthy Longevity Survey, 2018.

For peer review only

Setting People in urban and rural communities from 500 sample areas in 22 Chinese provinces.

Participants After exclusion, this study surveyed 9859 participants in the final analysis.

Primary and secondary outcome measures The main dependent variables were prevalence, awareness, and treatment of hypertension defined as (1) systolic blood pressure (SBP) ≥140 mmHg, diastolic blood pressure (DBP) ≥90 mmHg, or (2) taking antihypertensive drugs. Hypertension awareness was defined as a previous diagnosis of hypertension by a health professional, and hypertension treatment was defined as undergoing blood pressure treatment.

Results The prevalence of hypertension was lower among semi-urbanized adults than among non-urbanized rural adults (odds ratio [OR]=0.94, 95% confidence interval [CI]=0.90 to 0.99; P<0.05). The probabilities of awareness (OR=1.10, 95% CI=1.01 to 1.20; P<0.05) and treatment (OR=1.17, 95% CI=1.08 to 1.26; P<0.001) of hypertension were significantly lower among non-urbanized adults than among urban-born adults. Urbanization in eastern (OR=0.93, 95% CI=0.88 to 0.99; P<0.05) and western China (OR=1.11, 95% CI=1.01 to 1.23; P<0.05) was associated with the prevalence of hypertension. The urbanization level was also associated with hypertension awareness and treatment in eastern (OR=1.17, 95% CI=1.04 to 1.32; P<0.01; OR=1.26, 95% CI=1.14 to 1.40; P<0.001), central (OR=1.31, 95% CI=1.05 to 1.63; P<0.05; OR=1.29, 95% CI=1.08 to 1.55; P<0.01), and western China

For peer review only

For peer review only - http://bmjopen.bmj.com/site/about/guidelines.xhtml

(OR=1.28, 95% CI=1.07 to 1.53; P<0.01; OR=1.34, 95% CI=1.15 to 1.57; P<0.001). The Blinder-Oaxaca decomposition suggested that approximately 42% and 39% of the urban-rural gap in hypertension awareness and treatment, respectively, could be attributed to coefficient difference.

Conclusions: Public health programs and policies for chronic diseases should adjust with urbanization and combine individual-centered strategies.

Strengths and limitations of this study

- Our large sample from 22 provinces in China can clearly reveal the attribution difference of hypertension between urban and rural communities.
- Combine hukou (China's unique household registration system), current residence, and birthplace to comprehensively show the level of urbanization.
- Blinder-Oaxaca decomposition is used to determine the extent of gap in prevalence, awareness, and treatment of hypertension between urban and rural.
- The major limitation of our study is that we could not make causal inferences because of the cross-sectional nature.

For peer review only

For peer review only - http://bmjopen.bmj.com/site/about/guidelines.xhtml

INTRODUCTION

Hypertension is a major risk factor of stroke, heart disease, and renal failure.[1] Approximately 7.3 million people worldwide annually die from hypertension.[2] Between 1975 and 2015, the number of hypertensive patients increased from 594 million to more than 1.1 billion, mostly in low-and middle-income countries (LMICs).[3] Hypertension is a silent killer that rarely manifests symptoms in its early stages. However, elevated blood pressure increases the risk of chronic diseases, and early awareness and treatment are key to reducing the risk.[4] Yet, the proportion of people who were diagnosed and treated in LMICs was reportedly lower than that of high-income countries.[5]

The Grand Challenges emphasized the need to study the impact of urbanization on hypertension.[6] One of the key drivers of urbanization in LMICs was rural-to-urban migration, which was accompanied by a sharp increase in the prevalence of chronic diseases due to socioeconomic development, changes in lifestyles, and inequalities in health care utilization between urban and rural areas.[7] Therefore, it is meaningful to assess the impact of rural-to-urban migration on hypertension owing to its huge public health consequences.

This is particularly true in China, which has experienced a period of rapid urbanization. The seventh national census report in 2020 recorded 375.18 million individuals, an increase of 69.75% over 2010.[8] Moreover, the national cross-sectional survey from 2012 to 2015 showed that the prevalence of hypertension in the

For peer review only - http://bmjopen.bmj.com/site/about/guidelines.xhtml

population aged >60 years was 53.2%.[9] Due to the imbalance between urban and rural development as well as the different levels of urbanization development in eastern, northeastern, central, and western China,[10 11] there are gaps in the awareness and treatment of hypertension. The Chinese government has made great efforts to fill these gaps by explicitly investing in human resources to improve the number of doctors in township health centers and other public health programs.[12] However, rural/urban disparities concerning health services still exist,[13] and the urban-rural gap among the eastern, northeastern, central, and western regions is gradually widening.[14]

Previous studies have assessed the relationship between urbanization and the prevalence, awareness, and treatment of hypertension according to socioeconomic status.[7 13 15] The results of these analyses have highlighted that education, income, and the proportion of insured urban adults were significantly higher than those of rural adults, and these factors were significantly positively correlated with the awareness and treatment of hypertension. Some studies have also estimated the temporal trend of the prevalence, awareness, and treatment of hypertension with the development of urbanization.[16 17] Furthermore, there is a strong body of evidence for an increased risk of hypertension in children and adolescents in eastern, central, and western China, showing differing socioeconomic profiles.[14] Studying the among between the prevalence, awareness, and treatment among hypertension and urbanization and making comparisons within different areas of urbanization development can provide

For peer review only - http://bmjopen.bmj.com/site/about/guidelines.xhtml

insight into the development of rural and urban areas and their respective impacts on hypertension.

In view of current research, available reports and publications have largely examined and investigated hypertension and urbanization, but the relationship between urbanization and the prevalence, awareness, and treatment of hypertension in China has not been fully studied. First, most previous studies were based only on hukou (China's unique household registration system) or current residence and did not show the characteristics of the floating population.[18] Second, in recent years, there have been few studies on the awareness and treatment of urbanization and hypertension in the context of different economic regions throughout China.[19] Third, although a study has used the Blinder-Oaxaca decomposition to explain urban-rural disparities in hypertension,[16] most studies did not accurately quantify the extent to which the gaps in prevalence, awareness, and treatment of hypertension can be explained by urban-rural differences. Therefore, in this study, the urbanization classification was used to show in detail the changes in socio-demographic characteristics caused by population mobility, and the Blinder-Oaxaca classification was used to evaluate the gaps in prevalence, awareness, and treatment of hypertension caused by urban and rural areas.

The main purpose of this study was to examine (1) whether the level of urbanization is associated with the prevalence, awareness, and treatment of hypertension in eastern, northeastern, central, and western China after controlling for

For peer review only - http://bmjopen.bmj.com/site/about/guidelines.xhtml

education and income and (2) whether there is a gap in prevalence, awareness, and treatment of hypertension between urban-born and non-urbanized rural adults, and (3) the extent to which any gaps in prevalence, awareness, and treatment of hypertension can be explained by urban-rural differences. To the best of our knowledge, this is the first study to comprehensively show the impact of rural-to-urban migration on the prevalence, awareness, and treatment of hypertension based on the socio-demographic characteristics of the floating population in China. We hypothesize that the research results will provide insights for countries undergoing rapid social transformation and urbanization to develop effective measures to reduce health disparities and achieve healthy aging.

METHODS

Sampling

In this study, we used secondary data from the 2018 Chinese Longitudinal Healthy Longevity Survey (CLHLS), a longitudinal population-based study of people aged > 65 years in China. This data was the most recently available and therefore best fits China's current population situation. [20] Using a multistage stratified proportional probability sampling design, approximately 16,000 older people in urban and rural communities were randomly selected from 500 sample areas in 22 provinces. This study was approved by the Biomedical Ethics Committee of Peking University, and all study participants signed an informed consent form. In total, 9,859 individuals

For peer review only - http://bmjopen.bmj.com/site/about/guidelines.xhtml

were found eligible for this study, excluding 493 participants who refused to answer, 103 participants with technical problems, 870 participants who answered "not applicable," and 4,549 participants with missing data (Figure 1).

Variables

The main dependent variables in this study were prevalence, awareness, and treatment. We defined hypertension as (1) systolic blood pressure (SBP) ≥140 mmHg, diastolic blood pressure (DBP) ≥90 mmHg, or (2) taking antihypertensive drugs.[21] After the study participant rested for at least 5 min, a research assistant measured the BP of the right arm twice using a mercury sphygmomanometer at 1-min intervals, and the mean of the two measurements was calculated. For bedridden participants, BP was measured in the reclining position. Hypertension awareness was defined as respondents knowing that they had been previously diagnosed with hypertension by a health professional, assessed by the question, "Have you been hospital diagnosed with hypertension?" Hypertension treatment was defined as taking blood pressure treatment, correlating with the question, "Whether you take antihypertensive drugs?"

The key independent variable was the urbanization type. Four types of urbanization were identified: (1) non-urbanized: individuals were born and lived in rural areas, and held the rural hukou all the time; (2) semi-urbanized: individuals were born in rural areas but had moved into and lived in urban areas while still holding the rural hukou; (3) fully urbanized: individuals were born in rural areas, had moved in and lived in urban areas, and obtained the urban hukou; and (4) urban-born:

For peer review only - http://bmjopen.bmj.com/site/about/guidelines.xhtml

individuals were born and lived in urban areas and held the urban hukou all the time.[22]

we adjusted for demographic characteristics, including age (elderly age [60–75 years], senile age [75–90 years], long-livers [>90 years]),[23] sex (male, female), marital status (married: married and living together; not married: widowed, divorced, separated, or never married), and geographical region. We divided the regions by their economic belt: eastern China (including Beijing, Tianjin, Hebei, Shanghai, Jiangsu, Zhejiang, Fujian, Shandong, Guangdong, and Hainan provinces); central China (including Shanxi, Anhui, Henan, Hubei, and Hunan provinces); western China (including Guangxi, Chongqing, Sichuan, and Shaanxi provinces); and northeast China (including Liaoning, Jilin, and Heilongjiang provinces).

Socioeconomic characteristics included education, income, BMI, and medical insurance. Education and income were continuous variables representing years of schooling and total household income in the previous year, respectively. BMI was calculated as weight in kilograms divided by height in meters squared and classified as overweight and obese (≥24 kg/m^2), or others (<24 kg/m^2).[24] Medical insurance comprised urban resident basic medical insurance (URBMI), urban employee basic medical insurance (UEBMI), new rural cooperative medical scheme insurance (NCMS), and others such as commercial medical insurance and government-free medical insurance.

For peer review only - http://bmjopen.bmj.com/site/about/guidelines.xhtml

Lifestyle behavior included smoking status, alcohol drinking, and exercise. Participants who answered "smoking now" were recognized as yes, as was alcohol use and regular exercise.

Statistical analysis

Data were analyzed using STATA 15.0 for descriptive analysis, logistic regression, and the Blinder-Oaxaca decomposition. Descriptive statistical data on hypertension prevalence, awareness, and treatment among adults of the four types of urbanization are presented proportionally, and the corresponding chi-square and p-values were used to check whether there were statistically significantly differences among adults in the four urbanization levels. Continuous variables in demographic, socioeconomic, and lifestyle behaviors were estimated by means of averages, and categorical variables by proportions. The chi-square test was used for dichotomous variables, and the t-test was used for continuous variables.

We used logistic regression to examine the differences among adults of the four types of urbanization in a multivariate analysis adjusted for confounding variables. Adjusted odds ratios (ORs) and p-values are reported. The model is specified as follows:

$$logit(P_i) = \ln(\frac{P_i}{1-P_i}) = \beta_0 + \beta_1 urbanization_i + \sum_1^n a_n X_{ni} \quad (1)$$

where P_i represents the probability of the prevalence, awareness, and treatment of hypertension. $ubanization_i$ represents the four types of urbanization: non-

For peer review only - http://bmjopen.bmj.com/site/about/guidelines.xhtml

urbanized, semi-urbanized, fully urbanized, and urban-born. β_0 is a constant term, the coefficients β_1 represent disparities among the four types of urbanization and X_{ni} are control variables. In addition, subgroup analyses were performed using a logistic model to examine the impact of urbanization on the prevalence, awareness, and treatment of hypertension stratified by region in China after controlling for education and income.

The Blinder-Oaxaca decomposition is a commonly used disparities decomposition method. Based on the above-mentioned logit regression, this study will further use the Blinder-Oaxaca decomposition to decompose the disparities in the prevalence, awareness, and treatment of hypertension between urban and rural areas into three parts: endowment effect, coefficient effect, and interaction effect (the effect caused by the endowment effect and the coefficient effect at the same time).[25 26]

The factors that affected the prevalence, awareness, and treatment functions of hypertension were estimated for the two sets of samples, and the regression coefficients of the estimated equation were obtained. The Blinder-Oaxaca decomposition of the differences between the two sets was calculated as follows:

$$\bar{y}_a - \bar{y}_b = (\bar{x}_a - \bar{x}_b)\beta_b + \bar{x}_b(\beta_a - \beta_b) + (\bar{x}_a - \bar{x}_b)(\beta_a - \beta_b) \quad (2)$$

Where α were the adults living in urban areas and b were the adults living in rural areas; $(\bar{x}_a - \bar{x}_b)\beta_b$ was the "endowments effect" (or "explained component"); and $\bar{x}_b(\beta_a - \beta_b)$ was the "coefficients effect" (or "the unexplained component").

For peer review only - http://bmjopen.bmj.com/site/about/guidelines.xhtml

$(\bar{x}_a - \bar{x}_b)(\beta_a - \beta_b)$ can be explained by the interaction between the endowment effect and coefficient effect.

Patient and public involvement

All data in this study were derived from the CLHLS dataset. The CLHLS was approved by the Ethical Review Committee of Peking University (IRB00001052–13074). All participants signed the informed consent at the time of participation. None of the patients or the public were involved in the design or planning of this study.

RESULTS

Descriptive results

The background characteristics of the participants are listed in Table 1. The prevalence, awareness, and treatment of hypertension in rural-born adults were significantly lower than those in urban-born adults (P<0.001). The descriptive results showed that non-urbanized, semi-urbanized, fully urbanized, and urban-born adults also differed significantly in all control variables (P<0.001), and years of education increased with the level of urbanization. The income of people born in cities was more than twice that of those who were non-urbanized and semi-urbanized. The BMI of people born in cities was the highest, and it increased with the level of urbanization. Rural-born adults participating in the NCMS accounted for approximately 70.2% of the sample.

For peer review only - http://bmjopen.bmj.com/site/about/guidelines.xhtml

Association of urbanization and the prevalence, awareness, and medical use of hypertension

We regressed the three binary variables (prevalence, awareness, and treatment) on demographic characteristics, socioeconomic characteristics, and lifestyle behavior, as shown in Table 2. After controlling for confounding variables, semi-urbanized adults had lower odds of hypertension than did non-urbanized adults (OR=0.94, 95% confidence interval [CI]=0.90 to 0.99; P<0.05). Urban-born adults were 10% (OR=1.10, 95% CI=1.01 to 1.20; P<0.05) more likely to be diagnosed with hypertension than the reference group (rural-born). There was a similar difference in treatment; urban-born adults were 17% (OR=1.17, 95% CI=1.08 to 1.26; P<0.001) more likely to be medicated than non-urbanized adults.

The risk of hypertension was 12% (OR=1.12, 95% CI=1.06 to 1.20; P<0.001) more likely in the senile age group than in the reference group (elderly age). Long-livers were less likely to be diagnosed (OR=0.84, 95% CI=0.77 to 0.91; P<0.001) or medicated (OR=0.85, 95% CI=0.79 to 0.91; P<0.001) for hypertension than were elderly patients. The association between geographical region and hypertension was significantly different. Specifically, participants living in central and western China had 89% (OR=0.89, 95% CI=0.86 to 0.92; P<0.001) and 93% (OR=0.93, 95% CI=0.91 to 0.96; P<0.001) lower odds of hypertension than participants in eastern China, respectively. Adults with UEBMI/URBMI had a higher probability of

For peer review only

awareness (OR=1.67, 95% CI=1.25 to 2.28; P<0.01) and treatment (OR=1.40, 95% CI=1.08 to 1.82; P<0.05).

Subgroup analysis

Table 3 lists the results of the stratified analyses. Regarding geographic location, the prevalence of hypertension decreased by a factor of 0.93 for each level of urbanization in eastern China (OR=0.93, 95% CI=0.88 to 0.99; P<0.05). Additionally, the relationship between urbanization and the prevalence of hypertension was significant in western China, and participants were 1.11 times more likely to develop hypertension for each level of urbanization (OR=1.11, 95% CI=1.01 to 1.23; P<0.05). No significant association was noted between urbanization and the prevalence of hypertension in northeastern or central China. The urbanization level was associated with hypertension awareness and treatment in eastern China (OR=1.17, 95% CI=1.04 to 1.32; P<0.01; OR=1.26, 95% CI=1.14 to 1.40; P<0.001), central China (OR=1.31, 95% CI=1.05 to 1.63; P<0.05; OR=1.29, 95% CI=1.08 to 1.55; P<0.01), and western China (OR=1.28, 95% CI=1.07 to 1.53; P<0.01; OR=1.34, 95% CI=1.15 to 1.57; P<0.001). No significant associations were noted between urbanization and hypertension awareness and treatment in northeast China.

Decomposition analysis

The decomposition results in Table 4 show that the probabilities of being hypertensive were 61.18% (95% CI=0.60 to 0.62, P<0.001) for urban adults and 61.11% (95% CI=0.60 to 0.63, P<0.001) for rural adults. Both endowment and

For peer review only - http://bmjopen.bmj.com/site/about/guidelines.xhtml

coefficient effects were significant. The results for awareness and treatment were somewhat different. The overall urban-rural differences in the predicted probabilities of awareness and treatment were 6.68% (95% CI=0.04 to 0.09, P<0.001) and 10.54% (95% CI=0.08 to 0.13, P<0.001), respectively. Both endowment and coefficient effects were significant in the logistic decompositions for awareness and treatment, although the former was more important. Approximately 2.82% of the total urban-rural difference in hypertension awareness could be attributed to the coefficient effect. The treatment findings were comparable. The total differences and differences attributed to the coefficient effect between urban and rural hypertension awareness and treatment were greater than those between urban and rural hypertension prevalence.

DISCUSSION

Our study of 9,859 adults in 22 diverse provinces in China demonstrated that urbanization was associated with the prevalence, awareness, and treatment of hypertension. The prevalence of hypertension in semi-urbanized adults was lower than that in rural adults. The proportion of awareness and treatment of hypertension among non-urbanized rural adults was lower than that among urban-born adults.

The prevalence of hypertension was lower in semi-urbanized adults than in rural adults. This was highly relevant, given that a large proportion of floating people who migrated from rural to urban areas are young, healthy laborers,[27 28] including the

For peer review only - http://bmjopen.bmj.com/site/about/guidelines.xhtml

younger-elderly population. Although some studies have examined the prevalence pf hypertension in correlation with urbanization,[15 29] they did not reveal the differences in demographic composition caused by urbanization in detail. In contrast, our study was based on the socio-demographic characteristics of the floating population, suggesting that the proportion of young-elderly semi-urbanized adults was large, so the prevalence of hypertension was low.

We also observed a significant association between the awareness and treatment of hypertension and years of education in urbanization. Although some studies have documented a positive impact of education on the awareness and treatment of hypertension,[30 31] few have considered the impact of education in the context of urbanization. We conclude that the higher the level of urbanization, the higher the level of education. Better education levels are often correlated with better family income. People with better educational levels may pay more attention to their health[32] and possibly have a higher healthcare expenditure budget. Therefore, improving the population education level, especially for rural adults, may have important practical and theoretical significance.

The prevalence of hypertension among older adults in eastern China was higher than that in the central and western China (Table 2), which is similar to the results of previous studies.[16 33 34] The economic development and the level of urbanization in eastern China is higher than those in central and western China.[35 11] Thus, the elderly living in eastern China may have high living standards, good living conditions, poor

For peer review only - http://bmjopen.bmj.com/site/about/guidelines.xhtml

eating habits, long-term intake of high-salt and high-oil foods such as meat,[36] plus the lifestyle is fast-paced and high-pressured,[37] making them more likely to suffer from hypertension. Although the level of urbanization in the western region has improved greatly, compared with the eastern region, there is a big gap in both the urbanization rate and urban infrastructure construction.[11] Moreover, the urbanization level of the provinces and regions within the western region is also very different, and this gap will continue to exist for some time. However, we also found that the higher the level of urbanization within eastern China itself, the lower the prevalence of hypertension, indicating that urbanization is a protective factor against hypertension. A possible reason for this is that eastern China is economically developed, and urbanization has entered a normal state. Residents with a high level of urbanization may focus on the prevention and care of chronic diseases, such as hypertension. Similar incidence characteristics of chronic diseases have been reported in developed countries, such as the United States,[38] reflecting the uneven distribution of medical resources between eastern and western China and between urban and rural areas.

The type of insurance a resident enrolls in also has a different effect on hypertension awareness and treatment. Urban insurance enrollees had higher awareness and treatment than rural insurance enrollees and uninsured adults, which is consistent with earlier findings.[39] However, most floating people can only participate in the NCMS because of China's unique household registration system (HRS, Hukou System). To receive full reimbursement, these floating people had to seek medical

For peer review only - http://bmjopen.bmj.com/site/about/guidelines.xhtml

care in their hometowns.[40] Previous studies have shown that participation in local medical insurance for floating people is more conducive to the management and control of hypertension.[41 42] Therefore, further efforts are needed to integrate the health insurance system into a uniform system, which is beneficial for the management of hypertension and reduces obstacles in the process of urbanization. We did not observe any association between commercial medical insurance and government-free medical insurance because there were only 406 other medical insurance cases documented in our study.

Strengths and limitations

Our study has several strengths, including a large sample size from 500 urban and rural communities across 22 provinces in China, making it a useful supplement to the elderly group data for future studies. Further, the inclusion of multiple provinces and communities increases the generality of the results, and the standardization and defining comprehensive and systematic information help to evaluate the results.

This study also has several limitations. First, we cannot make causal inferences since this study is cross-sectional by nature. However, the relationship between urbanization and hypertension has been confirmed in some longitudinal studies[16], and our sample is large and may be representative in China, and the research results may be credible. Second, this database is for people in China, and the results obtained can only be used in China and cannot be extended to other countries. However, it can

For peer review only - http://bmjopen.bmj.com/site/about/guidelines.xhtml

provide useful insights for countries that are experiencing an aging society, such as that of China.

Conclusion

Urbanization was significantly associated with the prevalence, awareness, and treatment of hypertension, especially between non-urbanized rural adults and urban-born adults, in a representative national population in China. Our findings indicate the role of urbanization that generates differences within demographic composition, education, regions, and health insurance in shaping the distribution of prevalence, awareness, and treatment of hypertension. Moreover, the results suggest that the relationship among hypertension prevalence, awareness, treatment, and urbanization varies within eastern, northeastern, central, and western China and that public health programs and policies for chronic diseases should be adjusted with the process of urbanization and combined with individual-centered strategies.

For peer review only

Abbreviations CLHLS: Chinese Longitudinal Healthy Longevity Survey; LMIC: low- and middle-income countries; BMI: body mass index; URBMI: urban resident basic medical insurance; UEBMI: urban employee basic medical insurance; NCMS: new rural cooperative medical scheme insurance；SD: Standard deviation

Acknowledgements We thank the Chinese Longitudinal Healthy Longevity Survey, which provided the data in this research.

For peer review only - http://bmjopen.bmj.com/site/about/guidelines.xhtml

Contributions QY: conceptualization, methodology, data analysis, and writing. GZ: methodology, conceptualization, writing with review, funding. All authors approved the final version of the paper.

Funding This study was supported by National Natural Science Foundation of China (71774102).

Patient consent for publication Written informed consent was obtained from participants.

Provenance and peer review Not commissioned.

Data availability statement Data are available in open research data service platform of Peking University. Data for this study were sourced from Chinese Longitudinal Healthy Longevity Survey (CLHLS) and available here: https://opendata.pku.edu.cn

Ethics approval and consent to participate The Chinese Longitudinal Healthy Longevity Survey (CLHLS) was approved by the Ethical Review Committee of Peking University (IRB00001052–13074). All participants signed the informed consent at the time of participation. The research was performed in accordance with the Declaration of Helsinki.

Competing interests The authors declare that they have no competing interests.

ORCID IDs

Qiutong Yu http://orcid.org/0000-0002-1758-0692

Genyong Zuo http://orcid.org/0000-0002-9305-9671

For peer review only

For peer review only - http://bmjopen.bmj.com/site/about/guidelines.xhtml

Table 1 Background characteristics of study participants aged 65 and over from Chinese Longitudinal Healthy Longevity Survey 2018 survey data (n=9859)

Variables	Rural-born			Urban-born	Total	P
	Non-urbanized	semi-urbanized	Fully-urbanized			
Prevalence, n (%)						<0.001
No	1609 (38.89)	1195 (41.90)	522 (37.10)	504 (34.45)	3830(38.84)	
Yes	2528 (61.11)	1657 (58.10)	885 (62.90)	959 (65.55)	6029(61.16)	
Awareness, n (%)						<0.001
No	420 (21.07)	255 (19.36)	102 (13.08)	69 (8.00)	846 (17.08)	
Yes	1573 (78.93)	1062 (80.64)	678 (86.92)	794 (92.00)	4107 (82.92)	
treatment, n (%)						<0.001
No	657 (33.17)	418 (31.98)	152 (19.56)	97 (11.23)	1324 (26.86)	
Yes	1324 (66.83)	889 (68.02)	625 (80.44)	767 (88.77)	3605 (73.14)	
Age, n (%)						<0.001
Elderly age	1045 (25.26)	689 (24.16)	257 (18.27)	467 (31.92)	2458 (24.93)	
Senile age	1491 (36.04)	1008 (35.34)	546 (38.81)	550 (37.59)	3595 (36.46)	
Long-livers	1601 (38.70)	1155 (40.50)	604 (42.93)	446 (30.49)	3806(38.60)	
Sex, n (%)						<0.001
Female	2403 (58.09)	1611 (56.49)	708 (50.32)	761 (52.02)	5483 (55.61)	
Male	1734 (41.91)	1241 (43.51)	699 (49.68)	702 (47.98)	4376 (44.39)	
Marital status, n (%)						<0.001
Not married	2401 (58.04)	1694 (59.40)	793 (56.36)	713 (48.74)	5601 (56.81)	
Married	1736 (41.96)	1158 (40.60)	614 (43.64)	750 (51.26)	4258 (43.19)	
Area, n (%)						<0.001
Eastern	1867 (45.13)	1198 (42.01)	762 (54.16)	1009(68.97)	4836 (49.05)	
Northeastern	118(2.85)	56(1.96)	143 (10.16)	121 (8.27)	438 (4.44)	
Central	1170 (28.28)	715 (25.07)	202 (14.36)	136 (9.30)	2223 (22.55)	
Western	982 (23.74)	883 (30.96)	300 (21.32)	197 (13.47)	2362 (23.96)	
Education, mean (SD) [a]	2.23(3.10)	2.17(3.12)	5.09(5.00)	7.72(5.21)	3.44(4.32)	<0.001
Income, mean (SD)	31000.21 (32620.93)	32741.37 (32764.62)	65050.23 (33761.20)	72712.83 (31715.16)	42553.08 (36874.99)	<0.001
BMI						<0.001
<24	2969 (71.77)	2057 (72.12)	862 (61.27)	845 (57.76)	6733 (68.29)	
≥24	1168 (28.23)	795 (27.88)	545 (38.73)	618 (42.24)	3126 (31.71)	
Somking, n (%)						<0.001
No	3468 (83.83)	2355 (82.57)	1257 (89.34)	1291(88.24)	8371(84.91)	
Yes	669 (16.17)	497 (17.43)	150 (10.66)	172 (11.76)	1488 (15.09)	

For peer review only

For peer review only - http://bmjopen.bmj.com/site/about/guidelines.xhtml

Drinking, n (%)						0.030
No	3508 (84.80)	2420 (84.85)	1224 (86.99)	1277(87.29)	8429 (85.50)	
Yes	629 (15.20)	432 (15.15)	183 (13.01)	186 (12.71)	1430 (14.50)	
Exercise regularly, n (%)						<0.001
No	2911 (70.36)	1896 (66.48)	545 (38.73)	551 (37.66)	5903 (59.87)	
Yes	1226 (29.64)	956 (33.52)	862 (61.27)	912 (62.34)	3956 (40.13)	
medical insurance [b], n (%)						
None	342(8.27)	251(8.80)	260 (18.48)	254 (17.36)	1107 (11.23)	
UEBMI/URBMI	249(6.02)	298 (10.45)	829 (58.92)	989 (67.60)	2365 (23.99)	<0.001
NCMS	3472 (83.93)	2255 (79.07)	167 (11.87)	87(5.95)	5981 (60.67)	
Others	74(1.79)	48(8.80)	151 (10.73)	133(9.09)	406(4.12)	

1 [a] SD: Standard deviation

2 [b] UEBMI/URBMI: urban resident basic medical insurance /urban employee basic medical insurance; NCMS: new rural cooperative medical scheme insurance; Others: commercial medical insurance and government free medical insurance.

5

6 Table 2 Multivariate logistic regression model to determine factors associated with prevalence, awareness, treatment among participants aged 65 years and above

Variables	Prevalence AOR (95%CI)	Awareness AOR (95%CI)	Treatment AOR (95%CI)
semi-urbanized	0.94 (0.90, 0.99) *	1.04 (0.95, 1.14)	1.02 (0.94, 1.10)
Fully-urbanized	0.97 (0.92, 1.03)	1.04 (0.94, 1.15)	1.09 (1.00, 1.19)
Urban-born	0.99 (0.94, 1.03)	1.10 (1.01, 1.20) *	1.17 (1.08, 1.26) ***
Age: Senile age	1.12 (1.06, 1.20) ***	1.02 (0.91, 1.14)	1.08 (0.98, 1.18)
Age: Long-livers	0.98 (0.94, 1.03)	0.84 (0.77, 0.91) ***	0.85 (0.79, 0.91) ***
Sex: Male	0.83 (0.75, 0.91) ***	0.81 (0.68, 0.98) *	0.74 (0.63, 0.87) ***
Married	0.91 (0.82, 1.00)	1.06 (0.88, 1.29)	1.12 (0.95, 1.31)
Region: Northeastern	0.98 (0.88, 1.09)	0.81 (0.67, 0.98) *	0.84 (0.71, 0.99) *
Region: Central	0.89 (0.86, 0.92) ***	0.98 (0.91, 1.05)	0.95 (0.90, 1.01)
Region: Western	0.93 (0.91, 0.96) ***	0.90 (0.86, 0.95) ***	0.88 (0.85, 0.92) ***
Education	1.01 (0.99, 1.02)	1.03 (1.00, 1.06) *	1.04 (1.02, 1.06) **
Income	1.00 (1.00, 1.00)	1.00 (1.00, 1.00) **	1.00 (1.00, 1.00) ***
BMI ≥24 (kg/m^2)	2.01 (1.83, 2.22) ***	1.43 (1.20, 1.70) ***	1.49 (1.28, 1.72) ***
Somking status: Yes	0.94 (0.83, 1.07)	1.05 (0.83, 1.34)	1.03 (0.853, 1.27)
Alcohol use: Yes	0.97 (0.86, 1.10)	0.66 (0.53, 0.83) ***	0.71 (0.58, 0.87) **
Exercise regularly: Yes	1.06 (0.97, 1.17)	1.01 (0.85, 0.19)	1.12 (0.97, 1.30)
UEBMI /URBMI [a]	1.10 (0.94, 1.29)	1.67 (1.25, 2.28) **	1.40 (1.08, 1.82) *
NCMS	1.10 (0.95, 1.27)	1.03 (0.79, 1.35)	1.00 (0.79, 1.26)

For peer review only - http://bmjopen.bmj.com/site/about/guidelines.xhtml

Others	0.89 (0.70, 1.14)	1.24 (0.77, 2.01)	0.94 (0.62, 1.41)
Constants	1.43 (1.17, 1.76) ***	4.08 (2.80, 5.92) ***	2.03 (1.47, 2.81) ***

CI, confidence interval; AOR, adjusted odds ratio. *** p < 0.001, ** p < 0.01, * p < 0.05

[a] UEBMI/URBMI: urban resident basic medical insurance /urban employee basic medical insurance; NCMS: new rural cooperative medical scheme insurance; Others: commercial medical insurance and government free medical insurance.

Table 3 Impact of urbanization on the prevalence, awareness and treatment of hypertension stratified by different regions in China after controlling education and income.

Variables	Prevalence	Awareness	Treatment
	AOR (95%CI)	AOR (95%CI)	AOR (95%CI)
Eastern	0.93 (0.88, 0.99) *	1.17 (1.04, 1.32) **	1.26 (1.14, 1.40) ***
Northeastern	0.97 (0.79, 1.18)	1.13 (0.79, 1.63)	1.00 (0.73, 1.37)
Central	1.05 (0.95, 1.17)	1.31 (1.05, 1.63) *	1.29 (1.08, 1.55) **
Western	1.11 (1.01, 1.23) *	1.28 (1.07, 1.53) **	1.34 (1.15, 1.57) ***

CI, confidence interval; AOR, adjusted odds ratio. *** p < 0.001, ** p < 0.01, * p < 0.05

Table 4 Blinder-Oaxaca decomposition results between urban and rural adults

	Prevalence	Awareness	Treatment
Predicted probability			
urban	61.18% (0.60, 0.62) ***	85.61% (0.84, 0.87) ***	77.37% (0.76, 0.79) ***
rural	61.11% (0.60, 0.63) ***	78.93% (0.77, 0.81) ***	66.83% (0.65, 0.69) ***
Difference in predicted probability			
Total difference	0.01% (-0.02, 0.02)	6.68% (0.04, 0.09) ***	10.54% (0.08, 0.13) ***
Due to endowments (explained)	2.31% (0.01, 0.04) **	3.52% (0.01, 0.06) **	6.56% (0.04, 0.09) ***
Due to coefficients (unexplained)	-0.02 (-0.04, -0.00) %*	2.82 (0.00, 0.05) % *	4.08% (0.01, 0.07) *
Due to interaction	0.07 (-0.02, 0.02) %	0.00% (-0.02, 0.03)	-0.00% (-0.03, 0.03)

CI, confidence interval. *** p < 0.001, ** p < 0.01, * p < 0.05

References

1 Stanaway JD, Afshin A, Gakidou E, et al. Global, regional, and national comparative risk

For peer review only - http://bmjopen.bmj.com/site/about/guidelines.xhtml

assessment of 84 behavioural, environmental and occupational, and metabolic risks or clusters of risks for 195 countries and territories, 1990–2017: a systematic analysis for the Global Burden of Disease Study 2017. *The Lancet* 2018;392(10159):1923-94.

2 World Health Organization. Hypetension and older people, 2017.

3 Zhou B, Bentham J, Di Cesare M, et al. Worldwide trends in blood pressure from 1975 to 2015: a pooled analysis of 1479 population-based measurement studies with 19·1 million participants. *The Lancet* 2017;389(10064):37-55.

4 Zhou B, Danaei G, Stevens GA, et al. Long-term and recent trends in hypertension awareness, treatment, and control in 12 high-income countries: an analysis of 123 nationally representative surveys. *The Lancet* 2019;394(10199):639-51.

5 Ikeda N, Sapienza D, Guerrero R, et al. Control of hypertension with medication: a comparative analysis of national surveys in 20 countries. *Bull World Health Organ* 2014;92(1):10-19C.

6 Daar AS, Singer PA, Persad DL, et al. Grand challenges in chronic non-communicable diseases. *Nature* 2007;450(7169):494-6.

7 Helble MC, Aizawa, Toshiaki. Urbanization and Inequality in Hypertension Diagnosis and Medication in Indonesia. *SSRN Electronic Journal* 2016

8 National Bureau of Statistics of the People's Republic of China. Interpretation of the Seventh National Census Communiqué, 2021. Available: http://www.stats.gov.cn/tjsj/sjjd/202105/t20210512_1817336.html. [accessed 17 Nov 2021].

For peer review only

For peer review only - http://bmjopen.bmj.com/site/about/guidelines.xhtml

9 Hua Q, Fan L, Li J, et al. 2019 Chinese guideline for the management of hypertension in the elderly. *J Geriatr Cardiol* 2019;16(2):67-99.

10 Wei H, Li L, Nian M. China's Urbanization Strategy and Policy During the 14th Five-Year Plan Period. *Chinese Journal of Urban and Environmental Studies* 2021;09(01)

11 Maimaitiming A, Xiaolei Z, Huhua C. Urbanization in Western China. *Chinese Journal of Population Resources and Environment* 2013;11(1):79-86.

12 Hesketh T, Zhou X. Hypertension in China: the gap between policy and practice. *The Lancet* 2017;390(10112):2529-30.

13 Aizawa T. Inequality in the treatment of diabetes and hypertension across residency status in China. *Ethn Health* 2021;26(4):512-29.

14 Fan Z, Liao Z, Zong X, et al. Differences in prevalence of prehypertension and hypertension in children and adolescents in the eastern, central and western regions of China from 1991-2011 and the associated risk factors. *PLoS One* 2019;14(1):e0210591.

15 Xiaohui H. Urban–Rural Disparity of Overweight, Hypertension, Undiagnosed Hypertension, and Untreated Hypertension in China. *Asia-Pacific Journal Of Public Health* 2008;20:159-69.

16 Li J, Shi L, Li S, et al. Urban-rural disparities in hypertension prevalence, detection, and medication use among Chinese Adults from 1993 to 2011. *Int J Equity Health* 2017;16(1):50.

17 Xing L, Jing L, Tian Y, et al. Urban-Rural disparities in status of hypertension in northeast China: a population-based study, 2017-2019. *Clin Epidemiol* 2019;11:801-20.

For peer review only - http://bmjopen.bmj.com/site/about/guidelines.xhtml

18 Bernabe-Ortiz A, Sanchez JF, Carrillo-Larco RM, et al. Rural-to-urban migration and risk of hypertension: longitudinal results of the PERU MIGRANT study. *J Hum Hypertens* 2017;31(1):22-28.

19 Li W, Gu H, Teo KK, et al. Hypertension prevalence, awareness, treatment, and control in 115 rural and urban communities involving 47 000 people from China. *J Hypertens* 2016;34(1):39-46.

[dataset]20 Peking University Center for Healthy Aging and Development. Data from: Chinese Longitudinal Healthy Longevity Survey. Peking University Open Research Data Repository, April 3, 2020. https://doi.org/10.18170/DVN/WBO7LK

21 World Health Organization. A Global Brief on Hypertension: Silent Killer, Global Public Health Crisis, 2013.

22 FAN Y VWQL. Childhood adversities, urbanisation and depressive symptoms among middle-aged and older adults: evidence from a national survey in China. *Ageing & Society* 2015;36:1031-51.

23 Dyussenbayev A. Age Periods Of Human Life. *Advances in Social Sciences Research Journal* 2017;4:258-63.

24 Chittaranjan S Yajnik JSY. Appropriate body-mass index for Asian populations and its implications for policy and intervention strategies. *The Lancet* 2004;363(9403):157-63.

25 Blinder AS. Wage Discrimination: Reduced Form and Structural Estimates. *The Journal of Human Resources* 1973;8:436-55.

26 R O. Male-Female Wage Differentials in Urban Labor Markets. *International Economic*

For peer review only - http://bmjopen.bmj.com/site/about/guidelines.xhtml

Review 1973;8:436-709.

27 Zhang W. A forecast analysis on world population and urbanization process. *Environment, Development and Sustainability* 2007;10(6):717-30.

28 Cheng Y, Rosenberg M, Winterton R, et al. Mobilities of Older Chinese Rural-Urban Migrants: A Case Study in Beijing. *Int J Environ Res Public Health* 2019;16(3)

29 Ahmed S, Tariqujjaman M, Rahman MA, et al. Inequalities in the prevalence of undiagnosed hypertension among Bangladeshi adults: evidence from a nationwide survey. *Int J Equity Health* 2019;18(1):33.

30 Daskalopoulou SS, Rabi DM, Zarnke KB, et al. The 2015 Canadian Hypertension Education Program recommendations for blood pressure measurement, diagnosis, assessment of risk, prevention, and treatment of hypertension. *Can J Cardiol* 2015;31(5):549-68.

31 Kaberi D RP, Luc P. BP, Robert R. Q. Managing hypertension: evidence supporting the 2013/2014 recommendations of the Canadian Hypertension Education Program. *CMAJ* 2015:116-19.

32 Leng B, Jin Y, Li G, et al. Socioeconomic status and hypertension: a meta-analysis. *J Hypertens* 2015;33(2):221-9.

33 Lu J, Lu Y, Wang X, et al. Prevalence, awareness, treatment, and control of hypertension in China: data from 1·7 million adults in a population-based screening study (China PEACE Million Persons Project). *The Lancet* 2017;390(10112):2549-58.

34 Kristi R DG, Paul M, Xigui W, Jing C,Guanyong H, Xiufang D, Paul K. Wheltona,and Jiang H. Geographic variations in the prevalence, awareness, treatment and control of

For peer review only

For peer review only - http://bmjopen.bmj.com/site/about/guidelines.xhtml

hypertension in China. *Journal of Hypertension* 2003;21:1273-80.

35 Yu X, Zhang W. All-cause mortality rate in China: do residents in economically developed regions have better health? *Int J Equity Health* 2020;19(1):12.

36 Wen F, Sun R, Ziaei SM. Research on the relationship between the imbalance of regional economic growth and the allocation of financial resources. *E3S Web of Conferences* 2021;275

37 Tin Tin Su1 HAM, Azmi Mohamed Nahar, Nurul Ain A. The effectiveness of a life style modification and peer support home blood pressure monitoring in control of hypertension: protocol for a cluster randomized controlled trial. *BMC Public Health* 2014;14:1-7.

38 Ostchega Y, Hughes JP, Zhang G, et al. Differences in Hypertension Prevalence and Hypertension Control by Urbanization Among Adults in the United States, 2013-2018. *Am J Hypertens* 2022;35(1):31-41.

39 Feng XL. Undiagnosed and Uncontrolled Chronic Conditions in China: Could Social Health Insurance Consolidation Make a Change? *Medical Care Research and Review* 2017;75:479-515.

40 Peiyuan Qiu YY, Juying Zhang and Xiao Ma. Rural-to-urban migration and its implication for new cooperative medical scheme coverage and utilization in China. *BMC Public Health* 2011;11:1-10.

41 Fang H, Jin Y, Zhao M, et al. Does Migration Limit the Effect of Health Insurance on Hypertension Management in China? *Int J Environ Res Public Health* 2017;14(10)

For peer review only - http://bmjopen.bmj.com/site/about/guidelines.xhtml

42 Li H, Zhu W, Xia H, et al. Cross-Sectional Study on the Management and Control of Hypertension Among Migrants in Primary Care: What Is the Impact of Segmented Health Insurance Schemes? *J Am Heart Assoc* 2019;8(16):e012674.

Figure legends/caption: Figure 1 Study flowchart of participants selection (aged 65years or over) from CLHLS 2018 survey data.

For peer review only

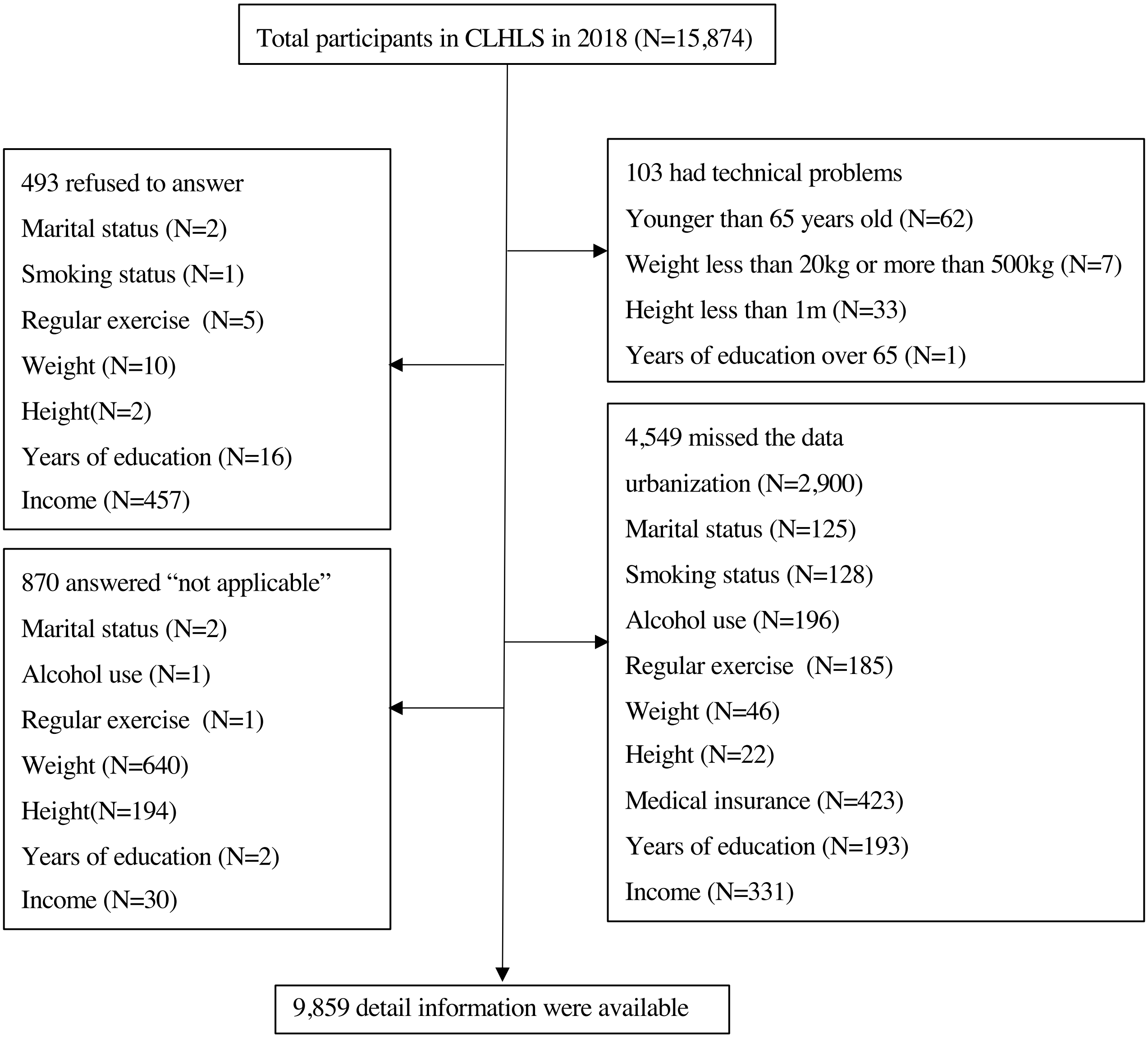

Figure 1 Study flowchart of participants selection (aged 65years or over) from CLHLS 2018 survey data.

STROBE Statement—Checklist of items that should be included in reports of ***cross-sectional studies***

	Item No	Recommendation	Page No
**Title and abstract**	1	(*a*) Indicate the study's design with a commonly used term in the title or the abstract	1
		(*b*) Provide in the abstract an informative and balanced summary of what was done and what was found	1-2
**Introduction**			
Background/rationale	2	Explain the scientific background and rationale for the investigation being reported	4-5
Objectives	3	State specific objectives, including any prespecified hypotheses	6-7
**Methods**			
Study design	4	Present key elements of study design early in the paper	7
Setting	5	Describe the setting, locations, and relevant dates, including periods of recruitment, exposure, follow-up, and data collection	7
Participants	6	(*a*) Give the eligibility criteria, and the sources and methods of selection of participants	7
Variables	7	Clearly define all outcomes, exposures, predictors, potential confounders, and effect modifiers. Give diagnostic criteria, if applicable	8-9
Data sources/ measurement	8*	For each variable of interest, give sources of data and details of methods of assessment (measurement). Describe comparability of assessment methods if there is more than one group	8-10
Bias	9	Describe any efforts to address potential sources of bias	7
Study size	10	Explain how the study size was arrived at	7
Quantitative variables	11	Explain how quantitative variables were handled in the analyses. If applicable, describe which groupings were chosen and why	8-10
Statistical methods	12	(*a*) Describe all statistical methods, including those used to control for confounding	10
		(*b*) Describe any methods used to examine subgroups and interactions	9-10
		(*c*) Explain how missing data were addressed	7
		(*d*) If applicable, describe analytical methods taking account of sampling strategy	
		(*e*) Describe any sensitivity analyses	/
**Results**			
Participants	13*	(a) Report numbers of individuals at each stage of study—eg numbers potentially eligible, examined for eligibility, confirmed eligible, included in the study, completing follow-up, and analysed	7
		(b) Give reasons for non-participation at each stage	/
		(c) Consider use of a flow diagram	24
Descriptive data	14*	(a) Give characteristics of study participants (eg demographic, clinical, social) and information on exposures and potential confounders	9
		(b) Indicate number of participants with missing data for each variable of interest	7
Outcome data	15*	Report numbers of outcome events or summary measures	8
Main results	16	(*a*) Give unadjusted estimates and, if applicable, confounder-adjusted estimates and their precision (eg, 95% confidence interval). Make clear which confounders were adjusted for and why they were included	12

For peer review only

		(*b*) Report category boundaries when continuous variables were categorized	12
		(*c*) If relevant, consider translating estimates of relative risk into absolute risk for a meaningful time period	/
Other analyses	17	Report other analyses done—eg analyses of subgroups and interactions, and sensitivity analyses	11
**Discussion**			
Key results	18	Summarise key results with reference to study objectives	15
Limitations	19	Discuss limitations of the study, taking into account sources of potential bias or imprecision. Discuss both direction and magnitude of any potential bias	18
Interpretation	20	Give a cautious overall interpretation of results considering objectives, limitations, multiplicity of analyses, results from similar studies, and other relevant evidence	18
Generalisability	21	Discuss the generalisability (external validity) of the study results	18
**Other information**			
Funding	22	Give the source of funding and the role of the funders for the present study and, if applicable, for the original study on which the present article is based	20

*Give information separately for exposed and unexposed groups.

Note: An Explanation and Elaboration article discusses each checklist item and gives methodological background and published examples of transparent reporting. The STROBE checklist is best used in conjunction with this article (freely available on the Web sites of PLoS Medicine at http://www.plosmedicine.org/, Annals of Internal Medicine at http://www.annals.org/, and Epidemiology at http://www.epidem.com/). Information on the STROBE Initiative is available at www.strobe-statement.org.

For peer review only
